# Patients with gastroenteric tumor after upper abdominal surgery were more likely to require rescue analgesia than lower abdominal surgery

**DOI:** 10.1186/s12871-022-01682-w

**Published:** 2022-05-23

**Authors:** Ting-Ting Li, Quan-Yuan Chang, Liu-Lin Xiong, Yan-Jun Chen, Qi-Jun Li, Fei Liu, Ting-Hua Wang

**Affiliations:** 1 Department of Anesthesiology, Institute of Neurological Disease, West China Hospital, Chengdu, 610041 Sichuan China; 2 Department of Anesthesiology, Southwest Medical University, Luzhou, 646000 Sichuan China; 3 Department of Anesthesiology, Affiliated Hospital of Zunyi Medical University, Zunyi, 563000 Guizhou China; 4 Traditional Chinese Medicine, Southwest Medical University, Luzhou, 646000 Sichuan China

**Keywords:** Dezocine, Abdominal operation, Postoperative pain, Emergency analgesia

## Abstract

**Objectives:**

To find out the reasons why patients still need to use rescue analgesics frequently after gastrointestinal tumor surgery under the patient-controlled intravenous analgesia (IV-PCA), and the different abdominal surgery patients using the difference of analgesics.

**Methods:**

A total of 970 patients underwent abdominal operation for gastrointestinal tumors were included. According whether patients used dezocine frequently for rescue analgesics within 2 days after surgery, they assigned into two groups: RAN group (Patients who did not frequently use rescue analgesia, 406 cases) and RAY group (Patients who frequently used rescue analgesia, 564 cases). The data collected included patient’s characteristics, postoperative visual analogue scale (VAS), nausea and vomiting (PONV), and postoperative activity recovery time.

**Results:**

No differences were observed in the baseline characteristics. Compared with the RAN group, patients in the RAY group had a higher proportion of open surgery, upper abdominal surgery, VAS score at rest on the first 2 days after surgery and PONV, and a slower recovery of most postoperative activities. Under the current use of IV-PCA background, the proportion of rescue analgesics used by patients undergoing laparotomy and upper abdominal surgery was as high as 64.33% and 72.8%, respectively. Regression analysis showed that open surgery (vs laparoscopic surgery: OR: 2.288, 95% CI: 1.650–3.172) and the location of the tumor in the upper abdomen (vs lower abdominal tumor: OR: 2.738, 95% CI: 2.034–3.686) were influential factors for frequent salvage administration.

**Conclusions:**

In our patient population, with our IV-PCA prescription for postoperative pain control, patient who underwent open upper abdominal surgery required more rescue postoperative analgesia.

## Introduction

Gastrointestinal tumor is the main malignant tumors in China, and surgery is the primary treatment. However, postoperative pain causes physical discomfort, psychological harm, prolong the patient’s length of hospital stay, and increase the cost of medical care. Adequate analgesia can alleviate postoperative pain of patients and contribute to their recovery [1–4]. Patient-controlled intravenous analgesia (IV-PCA) is one of the commonly used postoperative analgesia methods in clinical practice [5]. However, the current postoperative analgesia effect of IV-PCA was uneven, and rescue analgesia was often needed [6, 7]. Dezocine, as a partial μ-receptor agonist, a κ-receptor antagonist, and a norepinephrine and serotonin reuptake inhibitor [8], that produces a longer analgesic effect [9, 10], no significant inhibitory effect on respiratory system [11], and few side effects [12]. It was widely used in perioperative analgesia, especially in rescue analgesia [7]. However, there were no relevant analysis and research about rescue analgesia. In addition, its well known that pain characteristics such as type, location, intensity and duration vary considerably after different surgical procedures [13]. And as known to everyone involved in postoperative pain in major abdominal surgery, a laparoscopic abdominal surgery is less painful than open abdominal surgery and the upper abdominal surgery is more painful than lower abdominal surgery. But there was no literature detailing these differences. Therefore, we conducted this study to investigate the reasons that frequently use dezocine for emergency analgesia after open gastrointestinal tumor surgery in patients who had already used IV-PCA. At the same time, through the analysis of the use of rescue analgesics, to know what different in the gastrointestinal tumor surgery patients using the difference of analgesics.

## Methods and materials

### Patients and group

This retrospective investigation reviewed 970 patients who underwent abdominal operation at West China Hospital, Sichuan University between October 2017 and July 2018. All patients had a history of gastrointestinal neoplasms. The patients who included in this study were all received same IV-PCA for sustained analgesia within 3 days after surgery. IV-PCA was prepared by sufentanil 2 μg/kg + flurbiprofen ester 400 mg + dexmedetomidine 20 μg + methoxyclopramide hydrochloride 60 mg + appropriate normal saline, a total of 200 ml analgesic solution. Before using IV-PCA, patients will be guided the correct use of IV-PCA. After initiation of IV-PCA, patients will be received 2 ml/h of analgesic fluid (background dose was 2 ml/h). When the analgesic effect was not satisfactory, 0.5 ml analgesic solution can be added by self-pressing the button, and no analgesic fluid was given when pressing again within 15 min (additional dose was 0.5 ml/15 min). Unsatisfactory analgesia effect refers to the fact that the visual analogue scale (VAS) score (a range from 0 to 10) was greater than 3. The use of rescue analgesia in addition to PCA with the background infusion was recorded as an indicator of pain intensity and analgesia efficacy. When the patient’s pain could not be alleviated after two consecutive given additional dose, rescue analgesics were used for analgesia.

Inclusion criteria: 1) Age ≥ 18 years old. 2) Patients underwent abdominal operation. 3) American Society of Anesthesiologists (ASA) classification II - III. 4) Elective surgery.

Exclusion criteria: 1) Patients with severe liver, kidney, or blood system disorders. 2) New York Heart Association (NYHA) cardiac function grade II and above. 3) Patients with drug allergy. 4) Patients transferred to intensive care after surgery. 5) The TNM staging system in tumor patients exceeds stage III.

Patients were divided into two groups based on whether or not they had frequent used dezocine (used more than twice in total) within 2 days (d) after surgery: Rescue analgesia No (RAN) group (number (n) = 406, 41.86%) and Rescue analgesia Yes (RAY) group (*n* = 564, 58.14%).

### Ethic

All included data were collected from the medical record system of West China Hospital of Sichuan University. This study and the application for the exemption from informed consent both were approved by the Ethics Committee of West China Hospital, Sichuan University (Approval No. 2018306, Date of approval: 5 September 2018). All procedures in this study were in accordance with the ethical standards of the Helsinki declaration and the international ethical guidelines for human biomedical research.

### Data

1) The VAS score at 24 hours (h) and 48 h after surgery 2) The incidence of nausea, vomiting and dizziness at 24 h and 48 h after surgery. 3) The general characteristics, including gender, age, height, weight, body mass index (BMI). 4) Information about the disease, including the operation type (laparotomy, laparoscopic surgery, etc), tumor site (esophagus, stomach, jejunum, ileum, etc), main site of surgery (upper abdominal surgery or lower abdominal surgery. Upper abdominal gastrointestinal tumor surgery is mainly esophagus or gastrectomy, jejunum, ileum tumor resection. Lower abdominal surgery is mainly colon cancer and rectal cancer.), and operation time. 5) The use of intraoperative analgesics (sufentanil, parecoxib, flurbiprofen and dezocine). 6) The cumulative number of dezocine administered within 2 days after surgery. 7) The time between the end of the operation and the resumption of each activity (first anal exhaust, drinking water, getting out of bed, urinary catheter removal, peritoneal drainage tube removal, gastric tube removal, and length of hospital stays).

### Statistical analysis

Data were analyzed by SPSS software (version 21.0, SPSS Inc., Chicago, IL, USA). The qualitative variables were analyzed by Fisher test and presented as number with percentage. Under the normal distribution, the quantitative variables were analyzed by independent t test,and expressed as mean and standard deviation (SD). Binary logistic regression analysis was performed to assess the effect of factors on postoperative frequent use of rescue analgesia, and the results were expressed by odds ratio (OR) value and 95% confidence interval (95% CI). Furthermore regression analysis was performed according to whether VAS >  3 in the resting state 24 h after surgery and to whether nausea occurred 24 h after surgery. *p* <  0.05 was considered as statistically significant.

## Results

The patients who underwent abdominal gastrointestinal tumors operation in the West China Hospital of Sichuan University between October 2017 and July 2018 were selected. A total of 970 patients were included to analyze the factors influencing the frequent use of postoperative rescue analgesia and more than half of the patients in this study required frequent use of dezocine for emergency analgesia after the abdominal operation (564/970, 58.14%).

### Characteristics and perioperative information of patients

A total of 59.5% men and 40.4% women were included in the analyse. The overall patient’s age was 57.91 **±** 12.94 years-old (a range of 17–92 years), body weight was 60.44 **±** 10.34 kg (a range of 34-115 kg), height was 162.61 **±** 8.67 cm (a range of 64–182 cm), and BMI was 22.90 **±** 3.15 kg/m^2^ (a range of 14.19–38.97 kg/m^2^). There was no statistically significant difference in the characteristics of the two groups, including gender (*p* = 0.507), age (*p* = 0.682), height (*p* = 0.846), weight (*p* = 0.911), and BMI (*p* = 0.353) (Table 1).

**Table 1:** Characteristics and surgery information of patients (RAN vs RAY)

Group		RAN ***n =*** 406 (41.86%)	RAY ***n =*** 564 (58.14%)	***p*** value
**Characteristics of patients**				
Gender, n (%)	Male	236 (58.27%)	341 (60.46%)	0.507
	Female	169 (41.73%)	223 (39.54%)	
Age, years old, Mean ± SD		57.71 ± 13.11	58.06 ± 12.82	0.682
Age cohorts, years old, n (%)	≤ 40	33 (8.13%)	50 (8.88%)	0.421
	40–60	197 (48.52%)	249 (44.23%)	
	> 60	176 (43.35%)	264 (46.89%)	
Height, cm, Mean ± SD		162.53 ± 7.64	162.66 ± 9.30	0.846
Weight, kg, Mean ± SD		60.40 ± 10.02	60.47 ± 10.57	0.911
BMI, kg/m^2^, Mean ± SD		23.03 ± 3.04	22.81 ± 3.23	0.353
**Surgery information**				
The operation type, n (%)	Laparotomy	260 (70.65%)	469 (83.45%)	< 0.001^a^
	Laparoscopically surgery	104 (28.26%)	82 (14.59%)	
	Superficial abdominal wall surgery	3 (0.82%)	9 (1.0%)	
	Laparoscopic transfer to open surgery	1 (0.27%)	2 (0.36%)	
Tumor location, n (%)	Esophagus or stomach	76 (22.75%)	204 (41.89%)	< 0.001^a^
	Jejunum or ileum	8 (2.40%)	21 (4.31%)	
	Colon or rectum	250 (74.85%)	262 (53.80%)	

*Abbreviation*: *N =* Number(s), *%* = Percentage(s), *SD =* Standard deviation, *cm =* Centimeter(s), *kg =* kilogram(s), *BMI =* Body mass index, *m =* Meter(s), *h =* Hour(s)

^a^The difference between the two groups was statistically significant, *p* <  0.05

**Fig. 1:**
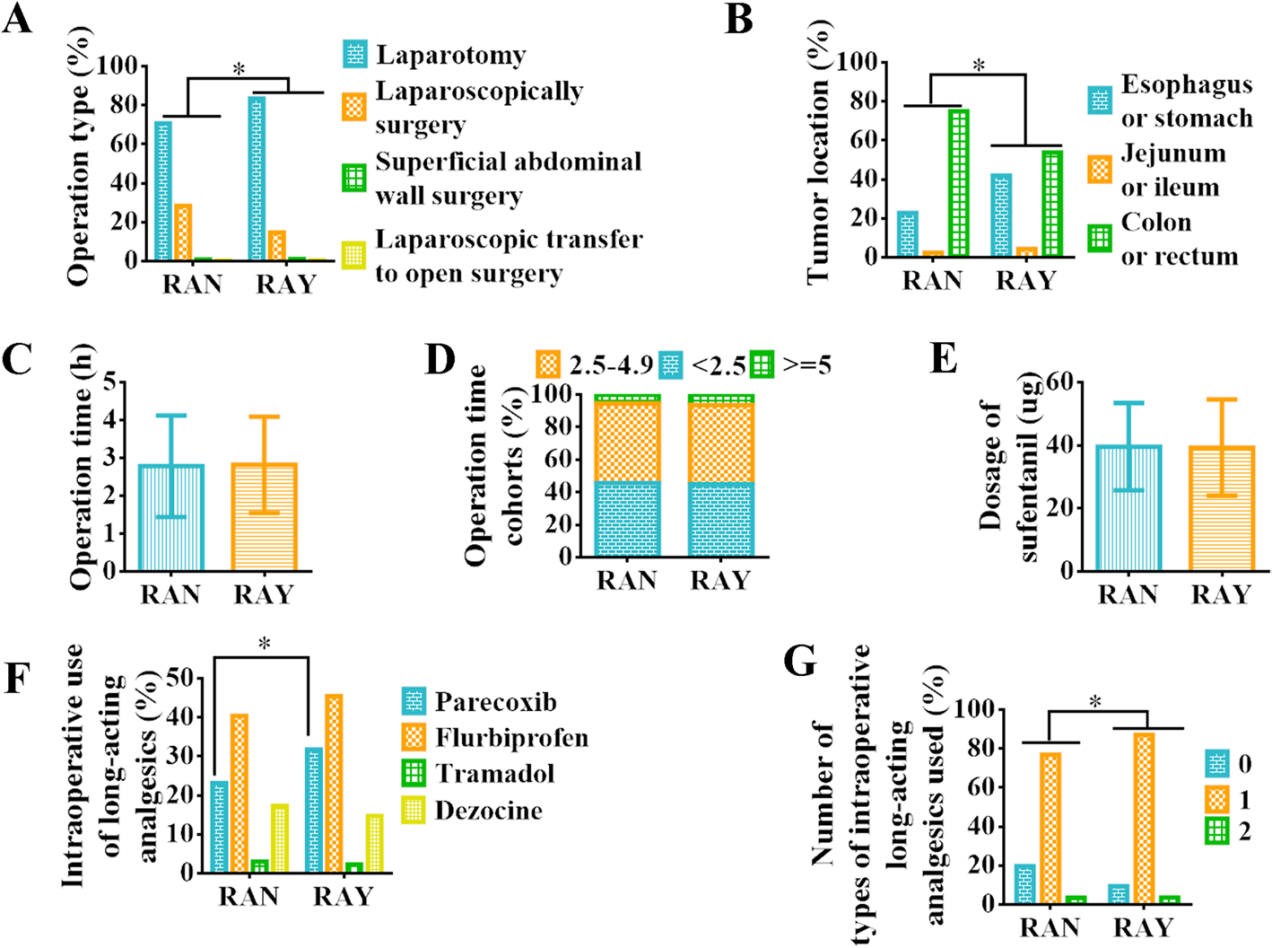
Comparison of patients’ intraoperative information. **A** The percentage of operation types (%). **B** The percentage of tumor locations (%). **C** The mean of operative time (h). **D** Percentage of operative time cohorts (%). **E** The mean of intraoperative dosage of sufentanil (ug). **F** Percentage of intraoperative use of various long-acting analgesics. **G** Percentage of types of long-acting analgesics used intraoperative analgesics (%). Abbreviation: % = percentage(s); h = hour(s); ug = microgramme. Note: *: The difference between the two groups was statistically significant, *p <* 0.05

Of all patients, 75.15% underwent laparotomy, 19.18% underwent laparoscopic surgery, 1.24% underwent superficial abdominal wall surgery, and 0.31% underwent laparoscopic transfer to open surgery. The proportion of postoperative rescue analgesia in patients undergoing open surgery was as high as 64.33%, which was higher than that of laparoscopic surgery (44.09%) (*p* <  0.001). Compared with the RAN group, patients in the RAY group had a greater proportion of open surgery (RAY: 469, 83.45% vs RAN: 260, 70.65%) and a smaller proportion of laparoscopic surgery (RAY: 82, 14.59% vs RAN: 104, 28.26%) (Fig. 1 A and Table 1, *p* <  0.001).

Of all patients, 42.14% underwent upper abdominal surgery (esophagus or stomach, jejunum or ileum) and 57.86% underwent lower abdominal surgery (sigmoid flexure, rectum). And the rate of postoperative rescue analgesia in patients undergoing upper abdominal surgery was 72.8%, higher than that in patients undergoing lower abdominal tumor surgery (49.5%) (*p* <  0.001). The RAY group had a relatively larger proportion of esophageal or stomach tumors (RAY: 204, 41.89% vs RAN: 76, 22.75%), jejunum or ileum tumors (RAY: 21, 4.31% vs RAN: 8, 2.40%), and a smaller proportion of colon or rectum tumors (RAY: 262, 53.80% vs RAN: 250, 74.85%) than the RAN group (Fig. 1 B and Table 1, *p* <  0.001).

There was no statistically significant difference in the duration of surgery between the two groups (RAY: 2.82 ± 1.27 vs RAN: 2.78 ± 1.34, *p* = 0.594, Fig. 1 C-D).

Except for parecoxib (RAY: 179, 31.74% vs RAN: 94, 23.15%, *p* = 0.004, Fig. 1 F), there was no statistically significant difference between the two groups in the analgesic drugs used during surgery (including the dosage of sufentanil (RAY: 39.29 ± 15.28 μg vs RAN: 39.57 ± 13.87 μg, *p* = 0.772, Fig. 1 E), flurbiprofen axel (RAY: 45.39% vs RAN: 40.39%, *p* = 0.131, Fig. 1 F), tramadol (RAY: 2.30% vs RAN: 2.96%, *p* = 0.544, Fig. 1 F), and dezocine (RAY: 14.72% vs RAN: 17.24%, *p* = 0.326, Fig. 1 F)). And a higher proportion of patients in the RAY group used long-acting analgesics during surgery than those in the RAN group (One kind analgesic: RAY: 491, 87.06% vs RAN: 312, 76.85%; Two kind analgesics: RAY: 20, 3.55% vs RAN: 14, 3.45%) (*p* <  0.001, Fig. 1 G).

### Postoperative pain status and adverse reactions

Within 2 days after surgery, the VAS scores in the RAY group were greater than those in the RAN group at rest (At 24 h after surgery: RAY: 1.35 ± 1.09 vs RAN: 1.18 ± 1.04, mean difference: 0.17, *p* = 0.012. At 48 h after surgery: RAY: 0.62 ± 0.77 **vs** RAN: 0.46 ± 0.66, mean difference: 0.16, *p* = 0.001). And there was no significant difference in VAS in active state (At 24 h after surgery: *p* = 0.579. At 48 h after surgery: *p* = 0.150) (Table 2).

**Table 2:** Evaluation of postoperative pain status and adverse reactions

Group		RAN ***n =*** 406 (41.86%)	RAY ***n =*** 564 (58.14%)	***p*** value
**24 h after surgery**				
VAS at resting, Mean ± SD		1.18 ± 1.04	1.35 ± 1.09	0.012 ^a^
	≤ 3, n (%)	396 (97.54%)	543 (9.28%)	0.355
	> 3, n (%)	10 (2.46%)	21 (3.72%)	
VAS at movement, Mean ± SD		2.84 ± 1.21	2.88 ± 1.29	0.579
	≤ 3, n (%)	303 (74.3%)	401 (71.10%)	0.243
	> 3, n (%)	103 (25.37%)	163 (28.90%)	
Dizzy, n (%)	Mild	26 (6.48%)	51 (9.19%)	0.472
	Moderate	21 (5.24%)	31 (5.59%)	
	Severe	9 (2.24%)	14 (2.52%)	
Nausea, n (%)		38 (9.43%)	81 (14.39%)	0.022 ^a^
Nausea associated with the gastric tube, n (%)		8 (1.99%)	24 (4.26%)	0.067
Vomiting, n (%)		4 (0.99%)	17 (3.02%)	0.042 ^a^
**48 h after surgery**				
VAS at resting, Mean ± SD		0.46 ± 0.66	0.62 ± 0.77	0.001 ^a^
	≤ 3, n (%)	398 (99.75%)	1 (0.25%)	0.645
	> 3, n (%)	558 (9.47%)	3 (0.53%)	
VAS at movement, Mean ± SD		1.72 ± 0.90	1.81 ± 1.08	0.150
	≤ 3, n (%)	385 (96.49%)	529 (94.30%)	0.127
	> 3, n (%)	14 (3.51%)	32 (5.70%)	
Dizzy, n (%)	Mild	16 (4.06%)	40 (7.18%)	0.124
	Moderate	10 (2.54%)	20 (3.29%)	
	Severe	4 (1.02%)	3 (0.54%)	
Nausea, n (%)		19 (4.82%)	43 (7.69%)	0.084
Nausea associated with the gastric tube, n (%)		3 (0.76%)	7 (1.25%)	0.536
Vomiting, n (%)		6 (1.52%)	8 (1.43%)	1.000

*Abbreviation: n* = Number(s), *% =* Percentage(s), *h =* Hour(s), *SD =* Standard deviation, *VAS =* Visual analogue scale

^a^The difference between the two groups was statistically significant, *p* <  0.05

At 24 h after surgery, the incidence of nausea was 12.32%, vomiting was 2.17%, and dizziness was 15.90%. And at 48 h after surgery, the incidence of nausea, vomiting and dizziness was 6.51%, 1.47%, and 9.78%, respectively. Compared with the RAN group, the proportion of nausea and vomiting on the first postoperative day increased significantly in the RAY group (Nausea: RAY: 81, 14.39% vs RAN: 38, 9.43%, *p* = 0.022; Vomiting: RAY: 17, 3.02% vs RAN: 4, 0.99%, *p* = 0.042), while the proportion difference on the second postoperative day was not statistically significant (Nausea: *p* = 0.084; Vomiting: *p* = 1.000). There was no significant difference in the proportion of postoperative dizziness between the two groups (At 24 h after surgery: *p* = 0.472; At 48 h after surgery: *p* = 0.124) (Table 2).

### Postoperative recovery information

Except for the removal of urinary catheter (*p* = 0.402) and gastric catheter (*p* = 0.265), there were statistically significant differences in the time of recovery of most activities (including first bowel movement (*p* <  0.001), drinking water (*p* = 0.003), getting out of bed (*p* = 0.001), and removal of abdominal drainage catheter (*p* = 0.014)) between the two groups after surgery (Table 3). Patients in the RAY group had significantly longer postoperative hospital stays (7.69 ± 3.33 d) than those in the RAN group (6.73 ± 2.63 d) (Table 3, *p* <  0.001). There was no significant difference in the quality of life assessment one month after surgery between the two groups (Table 3, *p* = 0.956).

**Table 3:** Postoperative recovery information

	RAN ***n =*** 406 (41.86%)	RAY ***n =*** 564 (58.14%)	Mean Difference	***p*** value
**Time from the end of surgery to the resumption of activities, h, Mean ± SD**				
First bowel movement	55.89 ± 31.56	65.91 ± 28.97	10.03	< 0.001 ^a^
Drinking water	46.46 ± 32.49	53.13 ± 33.77	6.67	0.003 ^a^
Get out of bed	49.10 ± 22.52	54.14 ± 25.51	5.04	0.001 ^a^
Pull out the gastrointestinal catheter	63.28 ± 58.61	69.36 ± 48.90	6.08	0.265
Pull out the urine catheter	94.83 ± 46.93	97.51 ± 45.38	2.68	0.402
Pull out the abdominal drainage catheter	129.79 ± 57.59	141.07 ± 63.96	10.03	0.014^a^
**Other information**				
Postoperative hospital stay, d, Mean ± SD	6.73 ± 2.63	7.69 ± 3.33	0.96	< 0.001^a^
Quality of life, Mean ± SD	12.72 ± 7.18	12.74 ± 3.97	0.02	0.956

*Abbreviation*: *n* Number(s), *%* Percentage(s), h Hour(s), *SD* Standard deviation, *d* Day(s)

^a^The difference between the two groups was statistically significant, *p* <  0.05

### Factors affecting postoperative used rescue analgesia

**Fig. 2:**
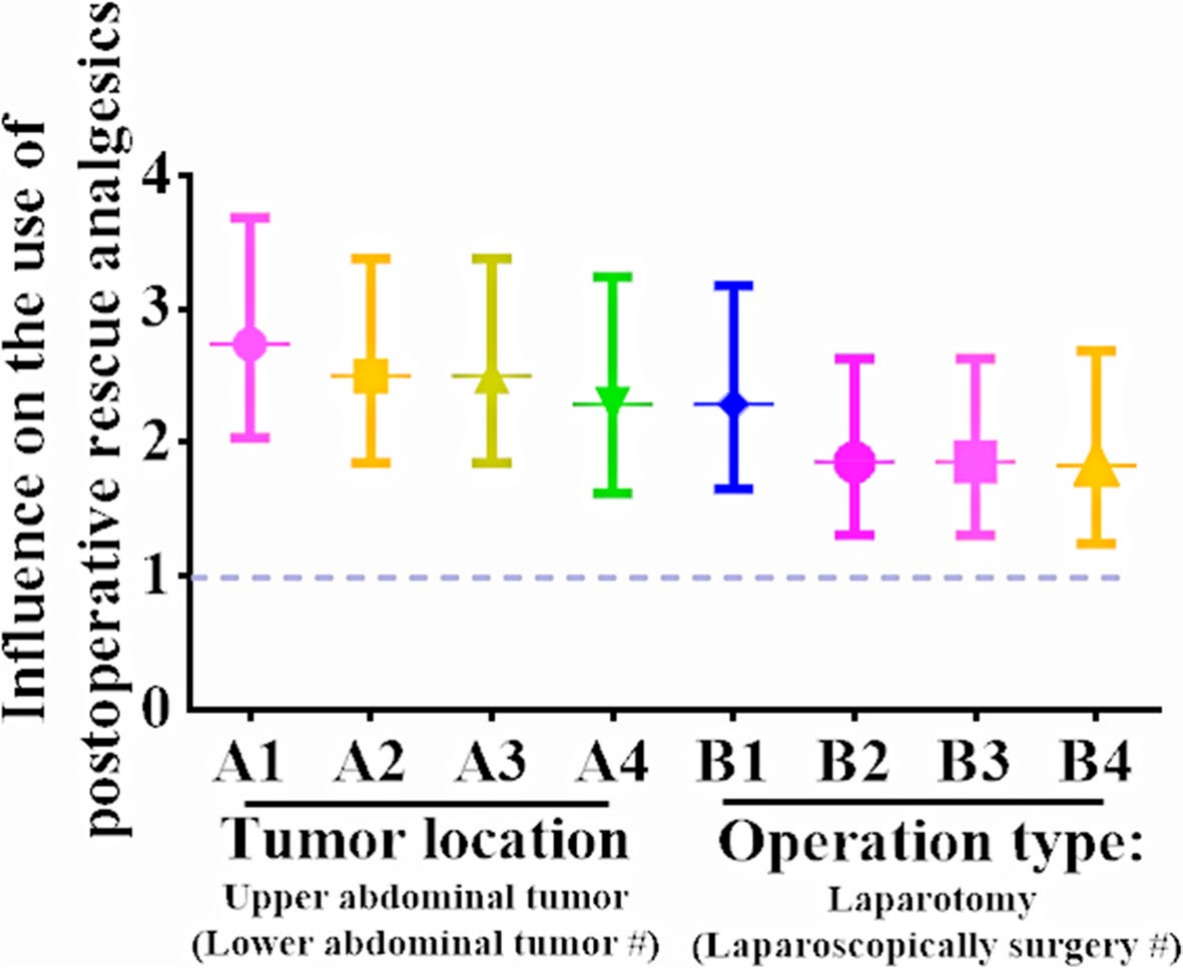
Effect of tumor location and type of operation on frequent use of rescue analgesics after operation. Tumor location: A1: Single-factor analysis. A2: Add parameter operation type. A3: Add parameters operation type and intraoperative long-acting analgesics used. A4: Add parameters gender, age, BMI, operation time, operation type and intraoperative long-acting analgesics used. Type of operation: B1: Single-factor analysis. B2: Add parameter tumor location. B3: Add parameters tumor location and intraoperative long-acting analgesics used. B4: Add parameters gender, age, BMI, operation time, tumor location and intraoperative long-acting analgesics used

The difference analysis showed that the tumor location was the influencing factor for the frequent use of postoperative rescue analgesia, and further regression analysis showed that the tumor location in the upper abdomen had a great influence on the patients (Table 4. Tumor location (1)). Furthermore, tumor sites were grouped according to their location in the upper or lower abdomen, and the results showed that patients undergoing surgery for upper abdominal tumors were more likely to require frequent postoperative rescue analgesia than those for lower abdominal tumors (Fig. 2 A1 and Table 4. Tumor location (2), OR = 2.738, 95% CI: 2.034–3.686, *p* <  0.001). And the other factor on the use of rescue analgesics was whether the operation was open or not (Fig. 2 B1 and Table 4, Type of surgery (1) and (2), All *p* <  0.001).

**Table 4:** Influencing factors to postoperative used rescue analgesia: Single-factor analysis

		RAN *n =* 406 (41.86%)	RAY *n =* 564 (58.14%)	*p* value	OR value	95% CI of OR	
Tumor location (1), n (%)	Esophagus or stomach ^a^	76 (22.75%)	204 (41.89%)	< 0.001 ^b^			
	Transverse colon	5 (1.50%)	7 (1.44%)	0.279	0.522	0.161	1.693
	Left colon	5 (1.50%)	20 (4.11%)	0.441	1.490	0.540	4.111
	Right colon	28 (8.38%)	39 (8.01%)	0.020 ^b^	0.519	0.299	0.901
	Sigmoid flexure	35 (10.48%)	29 (5.95%)	< 0.001 ^b^	0.309	0.177	0.539
	Rectum	177 (52.99%)	167 (34.29%)	< 0.001 ^b^	0.352	0.251	0.493
	Jejunum or ileum	8 (2.40%)	21 (4.31%)	0.959	0.978	0.416	2.301
Tumor location (2), n (%)	Lower abdominal tumor ^a^	240 (71.86%)	235 (48.25%)				
	Upper abdominal tumor	94 (28.14%)	252 (51.75%)	< 0.001 ^b^	2.738	2.034	3.686
Type of surgery (1), n (%)	Laparotomy ^a^	260 (70.65%)	469 (83.45%)	< 0.001 ^b^			
	Laparoscopically surgery	104 (28.26%)	82 (14.59%)	< 0.001 ^b^	0.437	0.315	0.606
	Superficial abdominal wall surgery	3 (0.82%)	9 (1.0%)	0.448	1.663	0.446	6.197
	Laparoscopic to open surgery	1 (0.27%)	2 (0.36%)	0.933	1.109	0.100	12.286
Type of surgery (2), n (%)	Laparoscopically surgery ^a^	104 (28.57%)	82 (14.88%)				
	Laparotomy	260 (71.43%)	469 (85.12%)	< 0.001 ^b^	2.288	1.650	3.172
The use of parecoxib sodium during surgery (Unused ^a^)		94 (23.15%)	179 (31.74%)	< 0.003 ^b^	1.543	1.154	2.064
Number of types of intraoperative long-acting analgesics used, n (%)	0 ^a^	80 (19.70%)	53 (9.40%)	< 0.001 ^b^			
	1	312 (76.85%)	491 (87.06%)	< 0.001 ^b^	2.375	1.633	3.456
	2	14 (3.45%)	20 (3.55%)	0.049 ^b^	2.156	1.002	4.639

Abbreviation: *OR =* Odds ratio, *CI* = Confidence interval, *%* = Percentage(s)

^a ^Reference variable

^b ^The difference was statistically significant, *p* < 0.05

Considering that the intraoperative use of parecoxib was one of the intraoperative use of long-acting analgesics, it was included together for binary logistic regression. The results showed that the number of long-acting analgesics used during operation had a greater impact on the need for rescue analgesia using dezocine after operation (OR = 2.170, 95% CI: 1.475–3.192, *p* <  0.001) (Table 5. Multivariate analysis (1)). Considering the relationship between the type of surgery (Fig. 2 B1, OR = 2.288, 95% CI: 1.650–3.172, *p* <  0.001) and the location of the tumor (Fig. 2 A1, OR = 2.738, 95% CI: 2.034–3.686, *p* <  0.001), both were included in the analysis, and the results showed that both were influential factors for postoperative rescue analgesia (Fig. 2 A2 and B2, Table 5. Multivariate analysis (2)). Whether the above three factors are included at the same time (Fig. 2 A3 and B3, Table 5. Multivariate analysis (3)) or the basic characteristics of the patients are included (Fig. 2 A4 and B4, Table 5. Multivariate analysis (4)), the influence of type of surgery (Fig. 2 B3, OR = 1.855, 95% CI: 1.309–2.630, *p* = 0.001. Figure 2 B4, OR = 1.829, 95% CI: 1.244–2.689, *p* = 0.002) and tumor location (Fig. 2 A3, OR = 2.498, 95% CI: 1.846–3.380, *p* <  0.001. Figure 2 A4, OR = 2.290, 95% CI: 1.619–3.240, *p* <  0.001) on the need for postoperative rescue analgesia still exists.

**Table 5:** Influencing factors to postoperative used rescue analgesia: Multiple-factor analysis

		-2LL	H-L test	***p*** value	OR value	95% CI of OR	
**Multivariate analysis (1)**		1294.233	0.958				
Number of types of intraoperative long-acting analgesics used, n (%)	0 ^a^			< 0.001 ^b^			
	1			< 0.001 ^b^	2.170	1.475	3.192
	2			0.130	1.834	0.836	4.022
The use of parecoxib sodium during surgery (Unused ^a^)				0.058	1.341	0.991	1.814
**Multivariate analysis (2)**		1049.825	0.952				
The operation type: Laparotomy (Laparoscopically surgery ^a^)				0.001 ^b^	1.855	1.309	2.630
Tumor location: Upper abdominal tumor (Lower abdominal tumor ^a^)				< 0.001 ^b^	2.498	1.846	3.380
**Multivariate analysis (3)**		1047.968	0.998				
The operation type: Laparotomy (Laparoscopically surgery ^a^)				0.001 ^b^	1.855	1.309	2.630
Tumor location: Upper abdominal tumor (Lower abdominal tumor ^a^)				< 0.001 ^b^	2.498	1.846	3.380
Number of types of intraoperative long-acting analgesics used, n (%)	0 ^a^			0.393			
	1			0.175	1.397	0.862	2.265
	2			0.583	1.273	0.538	3.012
**Multivariate analysis (4)**		886.667	0.101				
The operation type: Laparotomy (Laparoscopically surgery ^a^)				0.002 ^b^	1.829	1.244	2.689
Tumor location: Upper abdominal tumor (Lower abdominal tumor ^a^)				< 0.001 ^b^	2.290	1.619	3.240
The operation time, h				0.525	0.958	0.839	1.094
Age, years old				0.359	1.006	0.993	1.020
Gender: Female (Male ^a^)				0.816	0.962	0.697	1.330
BMI, kg/m^2^				0.629	0.988	0.939	1.039
Number of types of intraoperative long-acting analgesics used, n (%)	0 ^a^			0.450			
	1			0.351	0.749	0.409	1.374
	2			0.217	0.545	0.208	1.429

*Abbreviation*: -*2LL* = -2 log likelihood, *H-L* = Hosmer-Lemesho, *OR =* Odds ratio, *CI* = Confidence interval, *%* = Percentage(s)

^a ^Reference variable

^b ^The difference was statistically significant, *p* < 0.05

### Factors affecting pain at rest state 24 h after surgery

Since dezocine was used for rescue analgesia after surgery and the difference analysis showed that the major VAS difference between the two groups was 24 h after surgery at rest. The factors influencing the pain in resting state of patients 24 h after surgery were further analyzed. The results showed that the location of the tumor (*p* = 0.108), surgical style (*p* = 0.961) were no effect on postoperative pain score.

### Factors affecting postoperative nausea at 24 h after surgery

Univariate analysis showed that female (vs male, OR = 1.768, 95%CI: 1.202–2.600, *p* = 0.004), lower age (OR = 0.973, 95%CI: 0.950–0.996, *p* = 0.024), lower weight (OR = 0.975, 95%CI: 0.956–0.994, *p* = 0.011), 24 h VAS score (At rest: OR = 1.386, 95%CI: 1.178–1.632, *p* <  0.001. At movement: OR = 1.408, 95%CI: 1.216–1.630, *p* <  0.001) had an impact on postoperative nausea. The location of tumor (*p* = 0.448), surgical style (*p* = 0.361) and Intraoperative sufentanil dosage (*p* = 0.743) had no effect. The gender, age, height, weight and the 24 h VAS at rest were included in the analysis and the results showed that only the higher postoperative VAS score had an impact on the nausea of the patients on the first day after surgery (OR = 1.525, 95% CI: 1.256–1.853, *p* <  0.001). The gender, age, height, weight and the 24 h VAS at movement were included in the analysis and the results showed that female (vs male, OR = 1.974, 95%CI: 1.032–3.777, *p* = 0.040) and the higher postoperative VAS score (OR = 1.463, 95%CI: 1.225–1.747, *p* <  0.001) had an impact on the nausea of the patients on the first day after surgery.

## Discussion

A total of 970 patients who underwent abdominal surgery were collected in this study. They all received the same IV-PCA for continuous analgesia within 3 days after the operation. The patients were divided into two groups according to whether or not dezocine was frequently used for rescue analgesia 2 days after the operation. No differences were observed in the baseline characteristics. Compared with the RAN group, patients in the RAY group had a higher proportion of open surgery, a higher proportion of upper abdominal surgery, a higher proportion of intraoperative use of long-acting analgesics, a higher VAS score at rest on the first 2 days after surgery, a greater proportion of nausea and vomiting on the first day after surgery, and a slower recovery of most postoperative activities. Regression analysis showed that the location of the tumor, type of surgery and the number of long-acting analgesics used during the operation are the factors that affect the use of rescue analgesics after surgery. Among them, the use of rescue analgesics was higher possibility for open surgery and upper abdominal tumor surgery. The resting VAS score was higher in the first 2 days after surgery, and the proportion of nausea and vomiting on the first day after surgery was higher. Further logistic regression analysis showed that the postoperative pain of upper abdominal surgery was significantly higher than that of lower abdominal surgery. The effect of tumor location on postoperative pain remained after the inclusion of possible influencing factors.

### Patients who frequently used rescue analgesia had more adverse reactions and worse recovery after operation

Compared with patients who did not frequently use rescue analgesia, patients who frequently used rescue analgesia had a higher probability of nausea and vomiting on the first postoperative day and worse postoperative recovery (such as exhaust, drinking water, getting out of bed, and removal of abdominal drainage tube). Further regression analysis showed that higher postoperative VAS score was the influencing factor for postoperative nausea (OR = 1.463, 95%CI: 1.225–1.747). Repeated and frequent use of rescue analgesics was a sign of poor pain control. Poor pain control can lead to discomfort such as nausea and vomiting [14, 15]. In addition, poor pain control makes patients reluctant to move after surgery, thus affecting the recovery of postoperative function [16–18]. Patients who frequently used rescue analgesia had more postoperative adverse reactions and slower postoperative recovery.

### Open gastrointestinal tumor surgery has higher demand of postoperative rescue analgesia

The proportion of postoperative rescue analgesia in patients undergoing open surgery was as high as 64.33%, which was higher than that of laparoscopic surgery (44.09%). And regression analysis showed that the one of influence on the use of rescue analgesics was whether the operation was open or not. Patients undergoing open surgery were more likely to require rescue analgesia after surgery than patients undergoing laparoscopic surgery (OR = 2.288, 95% CI: 1.650–3.172). Shinichi Sakuramoto et al. performed laparotomy and laparoscopic surgery on 64 patients undergoing distal gastrectomy and found a significant reduction in postoperative analgesic use after laparoscopic surgery [19]. Caroline Lemoine et al. performed pyloromyotomy in infants and found that infants undergoing open surgery were more likely to experience postoperative pain in the ward than those undergoing laparoscopic surgery [20]. Similar results have been reported in many literatures [21]. These two methods have great differences in incision size, intraoperative blood loss and postoperative infection rate [22–24], which leads to a higher demand for rescue analgesia after open surgery.

### Upper abdominal tumor surgery has higher demand of postoperative rescue analgesia

The rate of postoperative rescue analgesia in patients undergoing upper abdominal surgery was 72.8%, apparently higher than that in lower abdominal tumor surgery (49.5%). Despite sufficient literature analysis, gender, age, BMI, and so on are predictors of postoperative pain. However, the literature does not provide a clear comparison of the effects of surgical incision or type on postoperative pain [25]. There was no significant difference between the upper and lower abdomen in the length of the surgical incision [26, 27]. Gastric cancer surgery has a wide range of operations, involving many organs and tissues [28–32], which was easy to damage the surrounding blood vessels and nerves, and increased postoperative pain. Compared with upper abdominal surgery, colon cancer surgery had a smaller extent of traction injury during surgery. These reasons may explain why upper abdominal surgery was more painful and required more pain relief than lower abdominal surgery.

### Strengths and limitations

Based on the analysis of the frequent use of rescue analgesics 2 days after gastrointestinal tumor surgery, this study found that, under the current use of IV-PCA background, the proportion of rescue analgesics used by patients undergoing laparotomy and upper abdominal surgery was as high as 64.33% and 72.8%, respectively. The probability of needing rescue analgesia after upper abdominal surgery was higher than that after lower abdominal surgery (OR = 2.290), and the probability of needing rescue analgesia after open surgery was higher than that after laparoscopic surgery (OR = 1.829). This study not only verified the clinical experience from the data, but also provided the utilization rate of postoperative rescue analgesia of laparotomy and laparoscopic surgery, of upper and lower abdominal surgery, providing guidance for the improvement of postoperative analgesia for gastrointestinal tumors in the next step. Of course, due to the complexity and particularity of clinical cases, this study also has certain limitations. For example, this research is a retrospective analysis and has certain flaws. The current conclusions may only apply to this article. In the later stage, further prospective studies and other research methods with higher levels of evidence are needed.

## Conclusions

In our patient population, with our IV-PCA prescription for postoperative pain control, patient who underwent open upper abdominal surgery required more rescue postoperative analgesia.

## Data Availability

The datasets used and/or analysed during the current study are available from the corresponding author on reasonable request.

## Abbreviations

IV-PCA: Patient-controlled intravenous analgesia
VAS: Visual analogue scale
ASA: American Society of Anesthesiologists
NYHA: New York Heart Association
OR: Odds ratio
CI: Confidence interval
BMI: Body mass index
